# Education as a Predictor of Chronic Periodontitis: A Systematic Review with Meta-Analysis Population-Based Studies

**DOI:** 10.1371/journal.pone.0021508

**Published:** 2011-07-21

**Authors:** Adrien Boillot, Bechara El Halabi, George David Batty, Hélène Rangé, Sébastien Czernichow, Philippe Bouchard

**Affiliations:** 1 Department of Periodontology, Service of Odontology, Rothschild Hospital, AP-HP, Paris 7 - Denis Diderot University, U.F.R. of Odontology, Paris, France; 2 Department of Nutrition, Ambroise Paré Hospital, University Versailles St-Quentin, Boulogne-Billancourt, France; 3 INSERM, U1018, Centre for Research in Epidemiology and Population Health, Villejuif, France; 4 Department of Epidemiology and Public Health, University College London, London, United Kingdom; Brigham & Women's Hospital, Harvard Medical School, United States of America

## Abstract

**Background:**

The impact of socioeconomic inequalities on health is well-documented. Despite the links of periodontal disease with cardiovascular diseases, adverse pregnancy outcomes and diabetes, no meta-analysis of socioeconomic variations in periodontal disease exists. This meta-analytic review was conducted to determine the extent to which education attainment influences risk of periodontitis in adults aged 35+ years in the general population.

**Methods:**

The authors searched studies published until November 2010 using EMBASE and MEDLINE databases. References listed were then scrutinised, our own files were checked, and, finally, we contacted experts in the field. The authors included only general population-based studies conducted in adults aged 35 years and more. All articles were blind reviewed by two investigators. In the case of disagreement, a third investigator arbitrated. Using PRISMA statement, two reviewers independently extracted papers of interest.

**Results:**

Relative to the higher education group, people with low education attainment experience a greater risk of periodontitis (OR: 1.86 [1.66–2.10]; p<0.00001). The association was partially attenuated after adjustment for covariates (OR: 1.55 [1.30–1.86]; p<0.00001). Sensitivity analyses showed that methods used to assess periodontitis, definition of cases, study country and categorization of education are largely responsible for the heterogeneity between studies. No significant bias of publication was shown using both the Egger (p = 0.16) and rank correlation tests (p = 0.35).

**Conclusions:**

In the studies reviewed, low educational attainment was associated with an increased risk of periodontitis. Although this evidence should be cautiously interpreted due to methodological problems in selected studies, efforts to eliminate educational inequalities in periodontitis should focus on early life interventions.

## Introduction

Chronic periodontitis is a bacterially induced inflammatory disease of the soft and hard tissues which support the tooth root [1]. It is caused by an accumulation of dental plaque, organized as a biofilm on the surface of the tooth crown and root [2], which lead to the destruction of periodontal connective tissue and alveolar bone; without treatment this results in tooth loss. Periodontitis may have long term consequences for health by increasing the risk of type 2 diabetes (T2D) [3], obesity [4], metabolic syndrome [5], cardiovascular diseases (CVD) and pulmonary diseases [6]–[8], adverse pregnancy outcomes [9], and premature mortality [8]. The condition is common, affecting up to 20% of the adult population in industrialised countries [10].

Chronic periodontitis is a multifactorial disease. However, the contribution of the different risk factors/indicators associated with the disease remains unclear. In addition to a role for oral hygiene, age, cigarette smoking [11] and T2D [3] in the aetiology of this disorder, a further risk factor may be poor social circumstances. There are good *prima facie* reasons to anticipate a relationship between low socio-economic status and chronic periodontitis, not least the strong link between socio-economic status and various health behaviours including smoking and diet [12]. Socio-economic adversity is also related to higher rates of a range of other important chronic diseases, including CVD [13] selected cancers [14], and mental illness [15].

Socio-economic status is typically characterised by income, occupational prestige, and educational achievement. Of these, educational attainment is stable across the adult life course [16] and, because it is usually peaks earlier in life than the other socio-economic indicators, it precedes the onset of most major chronic diseases so allowing investigators to separate cause from effect (reverse causality). Identifying socioeconomic at-risk subgroups for chronic periodontitis has the potential to enhance the effectiveness of preventative campaigns by focusing interventions, adopting specific strategies, reducing costs of public health decisions and obtaining active participation of target populations [17]. Given the strong associations described in the literature between chronic periodontitis and various systemic conditions (e.g, cardiovascular disease), such preventive programmes have also a global public health importance.

The most recent systematic review on socioeconomic variations in chronic periodontitis was published five six ago [18]. Accordingly, we undertook an updated systematic review of studies examining the association of educational attainment and chronic periodontitis in adults in the general population. Additionally, we aggregated the results from identified studies by conducting a meta-analysis.

## Methods

### Identification of Studies

Following the PRISMA statement [19], we performed a systematic review of all published observational studies conducted in individuals aged 35 and older, which investigated the association between level of education and risk of periodontitis. (Table S1)

Two independent reviewers trained in online article searches (AB and BEH) searched English language papers published before November 2010. We used a four-pronged approach to identifying papers. First, the MEDLINE and EMBASE searches were conducted using the MeSH and EMTREE. For MEDLINE, we used the following strategy: (“Periodontal diseases” [MeSH] OR “periodontitis” [MeSH] OR “chronic periodontitis” [MeSH] OR “periodontal attachment loss” [MeSH] OR “periodontal pocket” [MeSH] OR “alveolar bone loss” [MeSH]) AND (“education” [MeSH] OR “educational status” [MeSH] OR “marital status” [MeSH] OR “occupations” [MeSH] OR “income” [MeSH] OR “socioeconomic factors” [MeSH] OR “social class” [MeSH]). Limits: Human, English.. For EMBASE, search strategy was: (“periodontal disease” [EMTREE] OR “periodontitis” [EMTREE] OR “chronic periodontitis” [EMTREE] OR “periodontal pocket” [EMTREE] OR “alveolar bone loss” [EMTREE]) AND (“education” [EMTREE] OR “educational status” [EMTREE] OR “marital status” [EMTREE] OR “occupation” [EMTREE] OR “income” [EMTREE] OR “socioeconomics” [EMTREE] OR “social status” [EMTREE]). Limits: Human, English. Second, references listed in articles of interest were scrutinised. Third, we checked our own files. Fourth, we contacted selected experts in the field.

### Inclusion Criteria

We included all observational studies conducted in adults aged 35+ years in the general population with 1) case definition of periodontitis (probing or radiographic assessment, self-reported data) (Text S1), 2) a variable describing the level of education, 3) a quantitative assessment of the relationship between these two variables. Studies conducted in non-representative populations (e.g. samples of attendees in dental settings) were excluded to maximise external validity. When more than one report used the same data, studies resulting in lowest power were excluded except when prospective data were available. In the case of disagreement, the two investigators discussed the article and tried to find agreement. When consensus was not reached, a third investigator (P.B.) was involved until agreement was found.

### Data Abstraction

Two investigators (AB, BEH) independently extracted and tabulated basic information on each study: design, country where the study was conducted, sample size, number of cases, age range, definition of chronic periodontitis, partial or full-mouth recording, categories of level of education. Crude odds ratios (OR), or the data to compute them when crude estimates were unavailable, were also identified and extracted. Adjusted effect measures, including the covariates features in the multivariable model, were tabulated.

### Data Analysis

We transformed OR by taking their natural logarithms and calculating standard errors and corresponding confidence intervals [20]. Effects measures and their standard errors were pooled using an inverse variance method with random effects to account for statistical heterogeneity between studies. We calculated pooled OR and accompanying 95% confidence interval (95% CI) for the lowest versus the highest categories of education. Heterogeneity was assessed with the I^2^ statistic [21]. We carried out sensitivity analyses based on study design (longitudinal versus cross-sectional studies), year of publication (≤2000 versus >2000), region where the study was conducted (US versus non-US countries), sample size (≤1000 versus >1000), age (≥35 versus olders (≥65)), periodontal assessment method (partial-mouth versus full-mouth recording), definition of cases (combined clinical attachment loss (CAL) and periodontal probing depth (PPD) versus single probing measurement), number of categories of education (2 versus >2). Significance was set at p<0.05 and 95% CI were quoted throughout [18]. The Kappa statistic was used to assess interrater reliability between the two independent reviewers [22]. Publication bias was assessed by visually examining a funnel plot with asymmetry being formally assessed with both the Egger test [23] and the rank correlation test [24]. The data were analysed using Review Manager (RevMan, version 5.0, The Cochrane Collaboration, 2008). Bias of publication was measured using CMA (Comprehensive Meta Analysis, version 2.2.055, Biostat, 2010).

## Results

### Study characteristics

**Figure 1:**
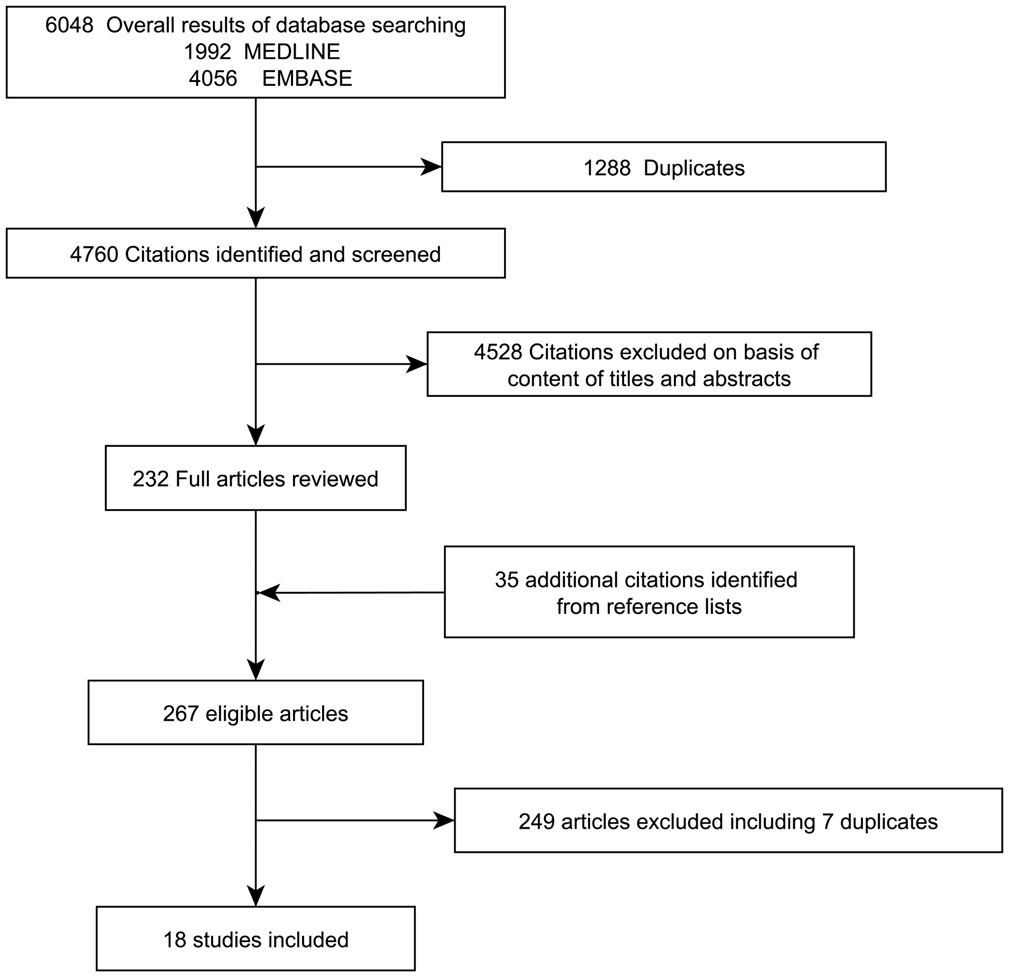
Flow chart for identifying eligible studies.

The electronic search yielded 6048 publications, including 1288 duplicates. From the 4760 potentially relevant articles identified, 4528 were excluded. Among the 232 remaining papers, hand-searching of references was performed, resulting in 35 additional papers. From the 267 eligible articles, 249 were excluded resulting in 18 studies, which met all the inclusion criteria and were included in the analyses (Figure 1) [25]–[42]. The kappa coefficient between examiners was 0.78 (0.75–0.80) demonstrating a substantial agreement [43]. Characteristics of the included studies are presented in Table 1–3. The combined population resulted in 40783 participants. Only two studies were longitudinal [31]–[32]. The included studies were reformed in the following countries (n = 10): Australia, Brazil, Canada, Denmark, Iran, Norway, Sweden, USA, Taiwan, and Thailand. Crude effect estimates were computed in ten studies [27], [29]–[31], [33], [34], [37], [39], [41]–[42]. Eight studies met criteria of inclusion, but were excluded because they were conducted in duplicate surveys [44]–[51].

**Table 1:** Characteristics of selected US cross-sectional studies in a meta-analysis of education level and chronic periodontitis.

Study name	Study size (of interest/original)	Age (yrs)	Study design	Outcome (No of cases in education groups of interest/Total No of cases)	Comparison groups for education	Variables in multiple adjustment
Beck et al. 1990, USA [25].	689/689	≥65	CS	At least 4 sites with CAL≥5 mm with at least one of those sites with PPD≥4 mm. n = 224/224.	< versus ≥12 years of education.	None.
Borrell et al. 2006, USA [26].	3,240/5,677	≥52	CS	At least two sites with CAL≥6 mm and at least 1 site with PPD≥5 mm. n = 545/963.	<High School versus ≥college.	Age, gender, center, neighbourhood socioeconomic score, income.
Dietrich et al. 2006, USA [28].	462/469	47–92	CS	At least one tooth with CAL and PPD≥5 mm. n = 86/86.	≤versus >High School.	None.
Dye et al . 2009, USA [29].	4,014/5,747	≥40	CS	At least one tooth with CAL≥3 mm and PPD≥4 mm. n = 843/1,063.	<High School versus ≥College.	Age, gender, smoking, race, diabetes, periodontal pathogens.
Famili et al. 2005, USA [30].	188/202	≥65	CS	More than 12 teeth with CAL>4 mm. n = 151/163.	≤versus >16 years of education.	None.
Phipps et al. 2009, USA [41].	672/1210	≥65	CS	30% or more of teeth examined with CAL≥5 mm. n = 248/463.	≤High School versus ≥Graduate School	None.

*CAL: Clinical Attachment Loss; CPITN: Community Periodontal Index of Treatment Needs; CS: Cross-Sectional; L: Longitudinal; PPD: Periodontal Pocket Depth; t0: Baseline data; Un: Unknown*.

**Table 2 pone-0021508-t002:** Characteristics of selected non-US cross-sectional studies in a meta-analysis of education level and chronic periodontitis.

Study name	Study size (of interest/original)	Age (yrs)	Study design	Outcome (No of cases in education groups of interest/Total No of cases)	Comparison groups for education	Variables in multiple adjustment
Brennan et al. 2007, Australia [27].	709/709	45–54	CS	2 or more sites with CAL≥5 mm and one or more sites with PPD≥4 mm. n = 139/139.	Primary, Secondary, Certificate versus Diploma or degree	None.
Hessari et al. 2007, Iran [33].	2,764/7,949	35–44	CS	At least one sextant with CPITN score 4 (PPD>5.50 mm). Men: n = 136/457. Women: n = 158/360.	Illiterate versus any university education.	None.
Krustrup et al. 2006, Denmark [34].	386/1052	35–44; 65–74	CS	At least one tooth with CAL≥6 mm. n = 22/50.	≤10 versus ≥15 years of education.	None.
Lai et al. 2007, Taiwan [35].	4,347/8,462	35–44	CS	CPITN score 4 (PPD>5,5 mm). n = Un/414.	≤ Junior High School versus ≥ College.	Age, gender, occupation.
Locker et al. 1993, Canada [36].	624/624	≥50	CS	Mean CAL≥3.83. n = Un.	≤ versus >High School.	None.
Mucci et al. 2004, Sweden [37].	14,736/26,690	≥42	CS	Self-reported. Diagnosed by a dentist to have periodontal disease or ever had tooth mobility. n = 2,956/5,527.	Elementary versus University	None.
Nicolau et al. 2007, Brazil [38].	224/224	39,01 (4.70)	CS	More than 42% of teeth with loss of attachment. n = 90/90.	≤ versus >4 years of education.	Age, smoking, plaque, emotionnal support, conditions during childhood.
Paulander et al. 2004, Sweden [39].	549/549	50 ; 55	CS	Highest 20% of the CAL distribution (mean CAL: 2.4–7.1). n = 110/110.	≤ versus >7 years of education.	None.
Peres et al. 2007, Brazil [40].	6,086/11,342	35–44	CS	At least one site with PPD≥4 mm and at least one site with CAL≥4 mm. n = 542/1,018.	≤4 versus ≥12 years of education.	Age, gender, race, income.
Torrungruang et al. 2009, Thailand [42].	453/453	39–59	CS	3 or more sites with PPD≥5 mm. n = 164/164.	≤ versus >High School.	None.

*CAL: Clinical Attachment Loss; CPITN: Community Periodontal Index of Treatment Needs; CS: Cross-Sectional; L: Longitudinal; PPD: Periodontal Pocket Depth; t0: Baseline data; Un: unknown*.

**Table 3:** Characteristics of selected longitudinal studies in a meta-analysis of education level and chronic periodontitis.

Study name	Study size (of interest/original)	Age (yrs)	Study design	Outcome (No of cases in education groups of interest/Total No of cases)	Comparison groups for education	Variables in multiple adjustment
Gilbert et al. 2005, USA [31].	559/560	≥45	L	At least one site with a 48-month worst attachment level 3 mm or more than the baseline worst attachment level on that same tooth. n = 123/123.	< versus ≥High School.	None.
Hansen et al. 1995, Norway [32].	81/81	35 (t0)	L	Increased in the number of C-scored quadrants (PTNS) from 1973 to 1988 (at least one site with PPD>5 mm). N = 16/16.	≤ versus >10 years of education.	Missing teeth, last dental visit, oral preventive behaviours, socioeconomic proxies, social class.

*CAL: Clinical Attachment Loss; CPITN: Community Periodontal Index of Treatment Needs; CS: Cross-Sectional; L: Longitudinal; PPD: Periodontal Pocket Depth; t0: Baseline data; Un: unknown*.

Four out of 18 studies did not use full-mouth clinical examination [29], [35], [37], [41]. Only six studies, exhibiting a quite different level of adjustment, gave adjusted effect measures [26], [29], [32], [35], [38], [40]. Two studies were performed on samples of male [28], [41]; whereas two other studies were performed on samples of female [30], [38]. One study has separated the outcomes for each gender [33]. Cases were defined by a combination of CAL and PPD conditions in six studies [25]–[29], [40]; by a single CAL criteria in seven studies [30], [31], [34], [36], [38], [39], [41]; by CPITN score (Community Periodontal Index of Treatment Needs) in two studies [33], [35]; and by self-reported assessment in one study (for more informations about periodontal indices, see supplemental material) [36]. No study with radiographic assessment met criteria of inclusion.

### Summary estimates

**Figure 2:**
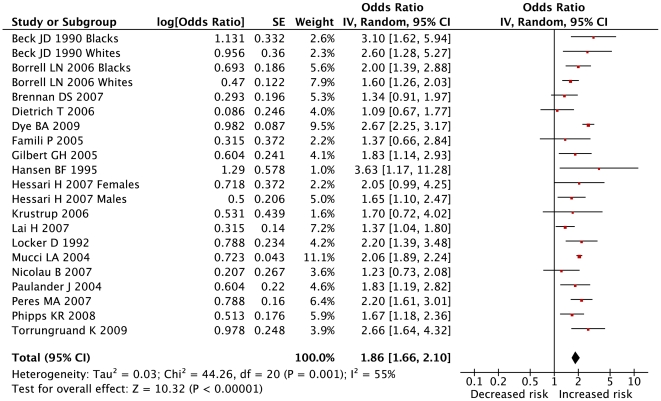
Results of primary meta-analysis: crude risk of periodontitis for individuals with lower education. Legend: Higher level of education as reference. Weights for individual studies calculated with random effects models and inverse variance method. The centre of each black square is placed at the point estimate, the area of the square is proportional to the sample size, and each horizontal line shows the 95% confidence interval for the estimate for each study. Pooled OR (95% CI): 1.86 (1.66–2.10). p<0.00001.

The summary estimates of OR for each study were pooled to give a total estimate of risk (Figure 2). The overall OR (95% CI) for chronic periodontitis was 1.86 (1.66 to 2.10), indicating an increased risk of periodontitis associated with a low level of education when compared with the highest level of education. The effect size among studies showed a moderate to substantial heterogeneity (I^2^ = 55%).

### Secondary analyses

Results of the primary analysis were not changed in the sensitivity analysis (Table 4). Two exceptions were the alternative meta-analysis including longitudinal studies and those conducted before year 2000, which enhanced the overall odds ratio (respectively 2.11 (1.22 to 3.63) and 2.57 (1.87 to 3.53)). Within the sensitivity analyses, we examined also various variables as potential sources of heterogeneity. There was evidence of significant heterogeneity across studies that may be explained by geographical differences (Non US: I^2^ = 42% versus US: I^2^ = 68%), the extent of the clinical assessment [partial-mouth (I^2^ = 89%) versus full-mouth (I^2^ = 22%)], and the definition of cases [clinical attachment loss and periodontal probing pocket combined (I^2^ = 74%) versus probing pocket depth solely (I^2^ = 0%)]. Other sources of heterogeneity have to be interpreted with caution because of the differences in the number of studies in each category. The sensitivity analyses with one by one exclusions showed that one study had a significantly effect on heterogeneity (Data not shown) [29]. When excluded, heterogeneity between studies was nearly non significant (p = 0.04, I^2^ = 39%).

**Table 4:** Results of sensitivity analyses to investigate differences between studies included in the meta-analysis (random-effects model).

Included studies	No of studies	OR	95% CI	Residual heterogeneity (I^2^)
Study design				
Longitudinal [31], [32]	2	2.11*	1.22, 3.63	17%
Cross-sectional [25]–[30], [33]–[42]	16	1.85***	1.64, 2.09	58%
Baseline data				
≤2000 [25], [32], [36]	3	2.57***	1.87, 3.53	0%
>2000 [26]–[31], [33]–[35], [37]–[42]	15	1.79***	1.58, 2.03	61%
Study region				
US [25], [26], [28]–[30], [41]	6	1.89***	1.51, 2.37	68%
Non US [27], [31]–[40], [42]	12	1.83***	1.58, 2.11	42%
Sample size				
≤1000 [25], [27], [28], [30]–[32], [34], [36], [38], [39], [41], [42]	12	1.78***	1.49, 2.12	31%
>1000 [26], [29], [33], [35], [37], [40]	6	1.94***	1.65, 2.27	70%
Age (years)				
No limitation [26]–[29], [31]–[40], [42]	15	1.85***	1.62, 2.10	60%
Olders only (≥65) [25], [30], [41]	3	1.98**	1.41, 2.78	29%
Oral examination				
Partial-mouth [29], [35], [41]	3	1.85*	1.18, 2.91	89%
Full-mouth [25]–[28], [30]–[34], [36], [38]–[40], [42]	14	1.81***	1.59, 2.06	22%
Periodontal assessment				
CAL and PPD [25]–[29], [40]	6	1.93***	1.52, 2.46	74%
CAL or PPD [30]–[36], [38], [39], [41], [42]	11	1.70***	1.49, 1.94	0%
N° of classes for education				
2 [25], [27], [28], [30]–[32], [36], [38], [39], [42]	10	1.81***	1.46, 2.24	42%
2 [26], [29], [33]–[35], [37], [40], [41]	8	1.91***	1.66, 2.20	63%

*P<0.01.

**P<0.001.

***P<0.0001.

Higher level of education as reference. Random-effects model and inverse variance method.

**Figure 3:**
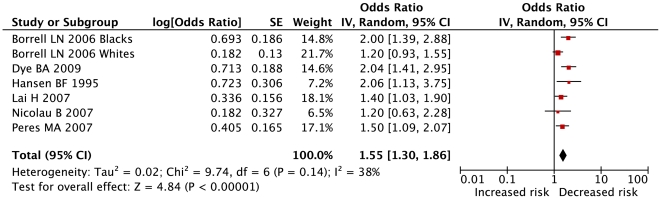
Results of primary meta-analysis: adjusted risk of periodontal diseases for individuals with low education. Legend: Higher level of education as reference. Weights for individual studies calculated with random effects models and inverse variance method. The centre of each black square is placed at the point estimate, the area of the square is proportional to the sample size, and each horizontal line shows the 95% confidence interval for the estimate for each study. Pooled OR (95% CI): 1.55 (1.30–1.86). p<0.00001.

When pooling studies with adjusted estimates, results were unchanged (OR = 1.55 (1.30 to 1.86); p<0.00001) but low heterogeneity between studies was found (I^2^ = 38%) (Figure 3). On the contrary, pooling crude estimates for same studies conducted to both an higher heterogeneity (I^2^ = 77%) and an higher risk estimate (OR = 1.88 (1.46 to 2.42)) (Data not shown). Funnel plot showed no asymmetry. No significant bias was shown using both the Egger (p = 0.16) and rank correlation tests (p = 0.35).

## Discussion

### Principal finding

The present analysis of studies conducted in the general population of adults reveals that individuals with a low level of education have an excess risk of chronic periodontitis when compared with adults with high level of education.

A number of mechanisms may explain this effect. Indirect mechanisms include the link between education and the two main risk factors for chronic periodontitis: smoking [52] and diabetes type 2 [53]. Lower educational attainment, which is a close correlate of IQ, may also directly lead to poorer coping strategies [54], higher BMI [55], lower levels of dental services use [56], low degree of periodontal health awareness [57], and irregular oral self-care practices [58], that in themselves are linked to poor oral hygiene habits may lead to higher levels of dental plaque [59]. Only one study which met our criteria of inclusion showed a significant decrease of the risk to develop chronic periodontitis in the low educated subgroup after adjustment for oral health behaviors [32].

Theories in social epidemiology could also explain such an association. The “pathway model” suggests circumstances in early-life influence social trajectories into and through adulthood, so increasing chronic disease risk [60]. Moreover, low education level is likely to lead to low prestige and low pay occupations, and residing in deprived area. The impact of environmental conditions on periodontal health has been widely described such that individuals living in a neighborhood in the most socially marginalised areas experience twice the risk of periodontitis relative to those in the most affluent [61].

### Exploration for heterogeneity

The pooled OR should be interpreted with caution, given the substantial heterogeneity detected between studies. Sensitivity analyses showed that several factors seem to account for this heterogeneity, including the region where the study was conducted, the extent of oral examination, the definition of cases and the number of categories for education level assessment. Taken together these factors might help us to improve our understanding of circumstances under which education interacts with periodontal health. In addition, the heterogeneity indicates that efforts should be made to adopt a standardised methodological approach when studying the association between periodontal diseases and socioeconomic factors.

Partial-mouth examinations may underestimate the prevalence of periodontal diseases [62], [63]. Additionally, methods used in partial-mouth examination are numerous. Both result in a lower heterogeneity between studies when excluding studies with partial-mouth examination. For same reasons, exclusion of studies using both CAL and PPD to define periodontitis results in a low heterogeneity between studies. As an illustration, heterogeneity between studies became non-significant when we conducted a one-study removed sensitivity analysis excluding Dye study (I^2^ = 39%) [29]. In this cross-sectional study using data from NHANES III survey, cases of periodontitis were defined as a combination of clinical attachment loss and probing pocket depth criteria after a full-mouth examination.

The impact of education on periodontal status does not seem the same according to the geographic area. Heterogeneity was higher for US studies than for studies conducted in other countries. One explanation might be that the US studies were not focused on same spatial units, several using census data, whereas others were conducted in counties or neighbourhoods.

Heterogeneity could also be due to differences in the classification of education attainment. In the sensitivity analyses, we compared studies with two classes of education with other studies. When only studies separating education level into two classes were included in the analysis, a lower heterogeneity was found. This may be due to the method we used. We compared individuals from extreme categories of education attainment. Including studies with more than two classes of education; we excluded all individuals with an intermediate level of education. Further, the classification of education attainment differs between studies. Some authors use corresponding diploma; other authors use years of schooling to categorize the level of education. Both resulted in a higher heterogeneity between studies with more than two classes of education.

Pooling of studies with adjusted estimates showed a decrease in heterogeneity between studies. Confounders such as race, diabetes or smoking are a source of heterogeneity between studies, and no adjustment result in an overestimated association between education and chronic periodontitis [18].

### Strengths and limitations

The present study has limitations inherent to meta-analyses. First, the results should be interpreted with caution and should not be considered as causal evidence because all studies were observational and most cross-sectional. Only two prospective studies met our criteria of inclusion [31]–[32]. Thus, associations are suggestive at best.

Secondly, there was significant heterogeneity between the included studies so overall conclusions must be regarded with caution. When we carried out sensitivity analyses to investigate possible sources of heterogeneity the results highlight a call for standardisation when studying social epidemiology in periodontal health, in particular concerning definition of cases. However, exclusion of only one study [29] resulted in a decrease of heterogeneity between studies, which became non significant (p = 0.04). Then, only six studies gave adjusted results for confounders [26], [29], [32], [35], [38], [40], and two studies were adjusted for smoking [29], [38], whereas it was described as a confounding factor [18].

Finally, because the physiopathology mechanisms for aggressive periodontitis differ from those involved in the development of chronic periodontitis [64] *a priori*, we elected not to include studies which captured the former outcome. This may have caused us to misclassify as unaffected some individuals with localized periodontitis. This is likely to have diluted the overall strength of the education-periodontal disease association.

However, our study has several strengths. It is the first, to our knowledge, including such a number of individuals (more than 40000), which quantifies the association between education attainment and periodontal diseases. The analysis is centred on chronic periodontitis, which is the most common form of periodontitis in adults. A previous systematic review has already shown a higher prevalence of periodontitis in a low socioeconomic group but the effect was not quantified [18]. The reason why no meta-analysis was performed in the above study maybe due (1) to the wide range of age (>19 year-old) that may include different diagnosis such as aggressive and chronic periodontitis, (2) to the difficulty to evaluate the socio-economic level in younger populations, and (3) to the low number of studies available when this review was conducted (our Medline search identified a nearly 60% increase in the number of studies from April 2004 to November 2010). Our search was conducted on multiple databases, and references listed in retrieved articles were adequately scrutinised according to the standards guidelines for systematic reviews. Finally, both Egger and rank correlation tests showed no significant bias of publication.

### Conclusions and implications

Results from our meta-analysis support the growing body of evidence of a socioeconomic gradient in oral health. The impact is first clinical to identify at-risk patients and adopt preventive attitudes for them, such as motivation to institute healthy behaviours (smoking cessation, oral hygiene instruction, regular dental visits…). This socioeconomic gradient in periodontal health may also have several implications for public health. The association between periodontal disease and other diseases that may compromise health condition emphasises the need to identify at-risk populations. This would permit to target public health actions aiming to prevent periodontal diseases. Identifying socioeconomic at-risk populations for oral diseases enhances the effectiveness of preventive campaigns by focusing interventions, adopting specific strategies and obtaining active participation of target populations. Moreover, because of the associations between education level and various risk factors/indicators for chronic periodontitis, educational attainment appears as a main target in preventing the development of chronic periodontitis. Finally, because of the relationships between chronic periodontitis and numerous systemic conditions, such approaches may lead to the reduction of the morbidity for other chronic diseases and, as a consequence, to decrease the overall healthcare costs.

To conclude, the effect of low education attainment on higher risk of periodontal diseases has a series of potential mechanisms. This notwithstanding, education may plausibly represent a main target for preventive programmes for periodontal diseases.

## Supporting Information

Table S1 **PRISMA checklist.**
(DOC)

Text S1 **Description of periodontal measurements.**
(DOC)
