# Supplementary material for: Education as a Predictor of Chronic Periodontitis: A Systematic Review with Meta-Analysis Population-Based Studies
Source: PLoS One. 2011 Jul 21;6(7):e21508. doi: 10.1371/journal.pone.0021508 (PMC3140980; doi:10.1371/journal.pone.0021508)
Supplement: Text S1 — Description of periodontal measurements. (DOC) [file pone.0021508.s002.doc]

**Clinical attachment loss (CAL) and probing pocket depth (PPD).**

Clinical attachment level (CAL) and probing pocket depth (PPD) are intra-oral measurements that evaluate periodontal destruction by using a straight probe graduated in millimetres. The probe indicates the distance between the base of the gingival pocket and the cemento-enamel junction (CAL) and/or the gingival margin (PPD).

PTNS and CPITN are both indices taking into account probing pocket depth.
